# Prospectus

**Published:** 1880-01

**Authors:** 


### PROSPECTUS.

The fixed purpose of the projectors of this journal to adhere with unvarying fidelity to independence in all matters appertaining to its conduct and management, renders it unnecessary to issue a lengthy prospectus of its objects and aims. With a few brief statements, therefore, it will be launched forth upon the sea of journalism to take its chances for public favor among the many craft now sailing more or less propitiously thereon. While it respectfully invites subscriptions from every quarter, it naturally looks for primary assistance from old friends and acquaintances. It will know no sectionalism, and, therefore, confidently expects to merit the friendship and good-will of those who *really* desire to sustain and keep from degradation to the level of the merest trade, the grandest and noblest calling known to humanity. From all such, subscriptions, contributions on subjects of interest to the profession, and advertisements are respectfully solicited. The experience of the senior editor, in the past, will be brought into activity in catering to the taste of its readers, and nothing will ever mar its pages not consistent with the standard that has been erected for its conduct and management. Being entirely unconnected with colleges and cliques, it will be bold, fearless and honest in speaking and upholding the right in *all* matters coming within its purview, and will scrupulously render even-handed justice to every one. All subjects of interest to Physicians, Obstetricians, Surgeons and Dentists will claim the attention of its editors and find space in its pages. To this end translations from the French and German periodicals will be introduced from time to time, and the English and American journals made tributary to its columns. All necessary arrangements have been made to *secure permanency* to the enterprise, and to keep it the independent echo and exponent of all that is new and good in the field of its labors. Exchanges of Medical, Dental and other scientific journals are respectfully requested from all parts of the world. Exchanges and books, except Dental, for notice or review. should be addressed to the senior editor, and Dental exchanges, subscriptions, advertisements, and *all* matters relating to the business management of the journal, should be sent invariably to the junior editor and proprietor.

